# Le carcinome parathyroïdien: à propos d’un cas et revue de la literature

**DOI:** 10.11604/pamj.2017.27.85.11584

**Published:** 2017-06-05

**Authors:** Naourez kolsi, Sondos Jellali, Jamel Koubaa

**Affiliations:** 1 Service ORL et CCF au CHU Fattouma Bourguiba, Monastir, Tunisie

**Keywords:** Parathyroïde, carcinome, hyperparathyroidie primaire, Parathyroid, carcinoma, primary hyperparathyroidism

## Abstract

Le carcinome parathyroïdien est une tumeur maligne, très rare, de la glande parathyroïde. Cliniquement, ce cancer se présente souvent par un tableau d'hyperparathyroïdie primaire sévère. Le diagnostic est histologique mais n'est pas toujours aisé. Le traitement est basé sur la chirurgie. Femme âgée de 59 ans, aux antécédents d'hypertension artérielle, et de lithiases rénales récidivantes, consultait pour des douleurs osseuses diffuses avec asthénie. L'examen du cou a trouvé une tuméfaction basi-cervicale dure et à bord inférieur non palpable. A la biologie: hypercalcémie à 4,1 mmol/l, une hyperparathyroïdie avec valeur de parathormone (PTH) très élevée à 1088 pg/ml soit 13 fois la normale. La scintigraphie au Technétium-99m-sestamibi a montré une plage de fixation anormale de MIBI en projection de la parathyroïde inférieure gauche. Une parathyroïdectomie inférieure gauche, avec évidement médiastino-récurrentiel homolatéral ont été réalisés. Les suites opératoires étaient marquées par la normalisation de la calcémie et de la PTH. L'anatomopathologie était en faveur d'un carcinome parathyroïdien. Le diagnostic de carcinome parathyroïdien est généralement établi sur la conjonction de signes radiologiques biologiques et histologiques. La gravité de cette pathologie est due à l'hypercalcémie sévère et au risque de récidive et de métastases à distance justifiant la surveillance prolongée.

## Abstract (en)

Parathyroid carcinoma is a very rare malignant tumor of the parathyroid gland. Clinically, this cancer is characterized by the presence of severe primary hyperparathyroidism. Diagnosis is based on histological examination but is not always easy. Surgery is the treatment of choice. We report the case of a 59-year old woman with a personal history of arterial hypertension and of recurrent renal lithiasis, presenting with diffuse bone pain associated with asthenia. Neck examination showed hard basi-cervical swelling with nonpalpable lower edge. Laboratory tests showed hypercalcaemia 4.1 mmol/l, hyperparathyroidism with very high parathyroid hormone (PTH) value (1088 pg/ml or 13 times normal). Technetium-99m-sestamibi scintigraphy showed fixed MIBI abnormality in the projection of the left inferior parathyroid. The patient underwent left inferior parathyroidectomy, with ipsilateral mediastinal-recurrent nerve dissection. The postoperative course was marked by normalization of plasma calcium and THP. Anatomopathological examination showed parathyroid carcinoma. The diagnosis of parathyroid carcinoma is generally based on the combination of biological, radiological and histological signs. The severity of this pathology is due to severe hypercalcaemia and to the risk of recurrence and distant metastases, justifying prolonged monitoring.

## Introduction

Le carcinome parathyroïdien est une tumeur maligne très rare de la glande parathyroïde, qui représente moins de 0,005% de tous les cancers, et moins de 1% des étiologies des hyperparathyroïdies primaires [1-4]. Cette maladie a été décrite pour la première fois en 1904 [5]. Cliniquement, ce cancer se présente souvent par un tableau d'hyperparathyroïdie primaire avec hypercalcémie sévère [4, 5]. Ce cancer ne présente aucune spécificité clinique ni biologique par rapport à l'adénome parathyroïdien ce qui expose à des difficultés diagnostiques. Par ailleurs, son diagnostic histologique n'est pas encore aisé, surtout en l'absence de métastases ou d'envahissement des organes de voisinage [5, 6]. Notre objectif est de chercher les particularités diagnostiques de ce cancer et de préciser ses modalités thérapeutiques à travers notre observation et une revue de la littérature.

## Patient et observation

**Figure 1:**
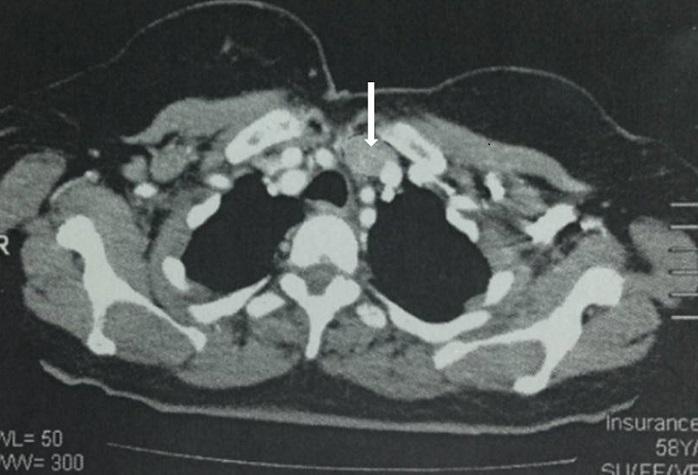
TDM cervico-thoracique en coupe axiale après injection de PDC montrant une masse hypodense (fleche) plongeant en rétrosternal, faiblement rehaussée, mesurant 20x36 mm, siégeant en dedans de la veine jugulaire interne et en avant de l’artère carotide commune gauche

**Figure 2:**
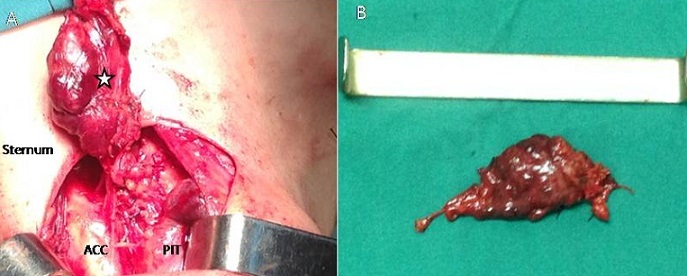
Aspect macroscopique peropératoire: (A) vue peropératoire lors de l'exérèse de la tumeur parathyroïdienne (étoile), ACC: artère carotide commune gauche, PIT: Pole Inférieur de la glande Thyroïde; (B) pièce opératoire de parathyroïdectomie inférieure gauche

Femme âgée de 59 ans aux antécédents d'hypertension artérielle, de troubles anxieux, et de lithiases rénales droites récidivantes depuis 30 ans, traitée par lithotripsie extracorporelle, avait des douleurs osseuses diffuses dans un contexte d'altération de l'état général. L'examen du cou trouvait une tuméfaction basi-cervicale antérieure latéralisée à gauche, dure, indolore, mobile à la déglutition, de 3 cm de grand axe à bord inférieur non palpable. Il n'y avait pas d'adénopathies cervicales palpables. La mobilité des cordes vocales était conservée. A la biologie: hypercalcémie à 4,1 mmol/l, une hyperparathyroïdie avec valeur de parathormone (PTH) très élevée à 1088 pg/ml soit 13 fois la normale, et des phosphatases alcalines élevées à 2870 UI/L. Les radiographies standards du squelette ont montré une déminéralisation osseuse diffuse surtout au niveau des os iliaques et des cols fémoraux avec résorption des houppes phalangiennes. L'échographie cervicale a objectivé une masse tissulaire hypoéchogène hétérogène en regard du pôle inférieur du lobe gauche de la thyroïde plongeant dans le médiastin antérosupérieur mesurant 36*19*16mm. Cette masse était hypervascularisée et avait un contact intime avec l'artère carotide commune homolatérale. La scintigraphie au Tc-99m-sestamibi a montré la présence d'une plage de fixation anormale de MIBI en projection du pôle inférieur du lobe gauche de la thyroïde, bas située, rétro-sternale évoquant un adénome au dépens de la parathyroïde inférieure gauche en position ectopique rétro-sternale. La tomodensitométrie (TDM) cervico-thoracique avait conclu à la présence d'une masse hypodense sous le lobe gauche de la thyroïde, bien limitée, mesurant 20*36 mm, siégeant en avant de l'artère carotide interne gauche, faiblement rehaussée après injection de Produit de contraste (PDC) (Figure 1). La TDM abdomino-pelvienne a montré une lithiase rénale droite de 16 mm sur un rein de néphropathie chronique sans dilatation pyélocalicielle, avec de multiples images de géodes sur le squelette osseux axial, de tailles variables. Au terme de ce bilan, Le diagnostic d'un adénome parathyroïdien a été évoqué et la chirurgie a été indiquée. Dans le cadre de préparation à la chirurgie, la patiente a eu une réhydratation par du sérum physiologique, avec administration de biphosphonates afin de corriger l'hypercalcémie. En per-opératoire, l'aspect macroscopique de la masse était fortement évocateur de malignité: c'était une masse dure, à limites irrégulières, et infiltrant les tissus avoisinants avec contact intime avec l'artère carotide commune (Figure 2). La masse était plongeante en rétrosternal, sans infiltration ni continuité avec le pole inférieur du lobe gauche de la glande thyroïde qui était macroscopiquement indemne. On a réalisée une parathyroïdectomie inférieure gauche et on a adressé la pièce opératoire pour examen anatomopathologique extemporané qui a évoqué la malignité d'où la décision de compléter par un évidement ganglionnaire médiastino-récurrentiel (EMR) homolatéral. Les suites opératoires immédiates étaient marquées par la normalisation des chiffres de la calcémie et de la PTH dosées à J1 postopératoire: calcémie à 2,25 mmol/l et PTH à 147 pg/ml puis à J3 postopératoire: calcémie à 2,45 mmol/l et PTH à 77 pg/ml. L'examen anatomopathologique définitif a conclu à un carcinome parathyroïdien qui consistait en une prolifération carcinomateuse sur 07 cm, encapsulée en partie, formée de lobules densément cellulaires, séparés les uns des autres par des septas fibrovasculaires. Les cellules tumorales avaient un aspect monomorphe. Elles avaient un cytoplasme abondant avec un noyau arrondi peu atypique. Les mitoses étaient estimées à 5 mitoses/10 CFG. Il existait par endroit des images d'effraction capsulaire et d'envahissement du tissu adipeux péri glandulaire avec des images d'emboles vasculaires tumoraux. Le curage ganglionnaire rmédiastino-récurrentiel homolatéral ne comportait pas de métastases ganglionnaires. L'examen de contrôle, 6 mois après la chirurgie, a noté une régression des douleurs osseuses, avec absence de signes cliniques et échographiques évoquant la récidive tumorale. Le contrôle biologique a trouvé une calcémie à 2,10 mmol/l et une PTH à 74pg/ml. Le recul actuel est 9 mois sans récidive.

## Discussion

Le carcinome parathyroïdien est une tumeur maligne très rare voire exceptionnelle. Elle représente moins de 0,005% de tous les cancers [1, 7]. C'est une néoplasie endocrinienne affectant environ 0,5 à 4% des patients souffrant d'une hyperparathyroïdie primaire avec une légère variation géographique: 1% en Europe et aux Etats-Unis et 5 % au Japon [2, 3, 6, 7]. Son incidence est extrêmement faible, environ 4 à 6 cas pour 10 millions d'habitants par an. La plus grande série publiée dans la littérature est celle de l'American National Cancer Data Base, a colligé 286 patients traités pour un carcinome parathyroïdien aux USA entre 1985 et 1995 [6, 8]. Le cancer de la parathyroïde pose une grande difficulté diagnostique et thérapeutique du fait de sa rareté et de l'absence de signes cliniques et paracliniques spécifiques, ce qui induit en erreur vers une hyperparathyroïdie primaire bénigne [2, 6, 9]. Son étiopathogénie, comme les autres cancers, est mal connue, mais il semble que certains facteurs génétiques et environnementaux interagissent d'une manière très complexe. L'irradiation du cou surtout à un âge jeune augmente le risque des néoplasies parathyroïdiennes [5, 6]. Certains mutations génétiques ont été rapportés dans le carcinome parathyroïdien, qui concernent principalement des oncogènes, des gènes suppresseurs des tumeurs : les pertes de 1p, 4q, et 13q, les gains de 1q, 9q, 16p et Xq ont été fréquemment observés dans le carcinome parathyroïdien [10]. D'autres études ont impliqué la surexpression de la cycline D1 dans la genèse du carcinome parathyroïdien puisqu' elle a été identifiée dans 91% de ces tumeurs [11]. Une forte association a été constatée entre le carcinome parathyroïdien et le syndrome 'hyperparathyroidism jaw tumor' qui est une maladie autosomique dominante rare où les individus atteints développent une hyperparathyroïdie primaire, des tumeurs fibro-osseuses mandibulaires, des tumeurs rénales et/ou utérines [12]. Lors de l'évaluation d'un patient ayant une hyperparathyroïdie primaire, il est difficile de prévoir, avant la chirurgie, de la nature bénigne ou maligne de la lésion en cause, du fait de l'absence de signes cliniques, et biologiques spécifiques permettant de différencier le carcinome de la parathyroïde de l'adénome parathyroïdien [6]. Tenant compte que la majorité des carcinomes parathyroïdiens sont sécrétants, le tableau clinique est souvent similaire à celui des affections parathyroïdiennes bénignes, Dominé par les signes d'hypercalcémie tels qu'une fatigue, malaise, faiblesse musculaire, amaigrissement, anorexie, des douleurs osseuses, des fractures pathologiques et des tumeurs ostéoclastiques kystiques dites tumeurs brunes, des troubles psychiatriques (dépression), et des symptômes digestifs (nausées, vomissements, douleurs abdominales, ulcère peptique, pancréatite et constipation). Des lithiases rénales avec des coliques néphrétiques peuvent encore s'associer [2-4]. Certains signes ne sont jamais habituels lors de l'hyperparathyroïdie bénigne et doivent attirer l'attention comme la dysphonie (orientant vers une paralysie récurrentielle due à l'invasion nerveuse locale), ainsi que la dysphagie, qui sont très suspectes de malignité [2]. La sévérité de l'hyperparathyroïdie primaire (taux élevé de calcémie et PTH) est un élément biologique de haute valeur, et doit faire suspecter la malignité [4, 6]. Il a été constaté que 65 à 75% des patients avec un cancer parathyroïdien avaient une calcémie supérieure à 3,50 mmol/L [10, 13]. Le taux de calcémie est en moyenne 10 fois la normale en cas de carcinome parathyroïdien par opposition à un taux de 2,6 fois la normale en cas d'hyperparathyroïdie primaire bénigne. Tous ces critères évoquant la malignité, ne font qu'orienter vers le cancer parathyroïdien sans pouvoir l'affirmer. Néanmoins, certains auteurs considèrent qu'un taux de PTH inférieur à 4 fois la normale rend le diagnostic de cancer parathyroïdien très peu probable [4, 6, 13]. Par ailleurs, en cas de carcinome parathyroïdien, on trouve souvent une phosphatase alcaline élevée, une hypophosphorémie et une acidose métabolique hyperchlorémique [4, 5].

Le diagnostic des carcinomes non sécrétants est encore plus difficile, vu l'absence de signes d'hyperparathyroïdie, contrairement aux carcinomes sécrétants. Ce type de cancer est extrêmement rare, souvent diagnostiqué tardivement lors d'une masse cervicale localement avancée [4, 6]. Quant au bilan radiologique, l'échographie cervicale et la scintigraphie au technétium-MIBI (méthoxy-isobutyl-isonitrile) ou Tc-99m-sestamibi sont bien informatives en matière de pathologie parathyroïdienne maligne [2, 3]. Le cancer de la parathyroïde apparait à l'échographie comme une lésion lobulée hypoéchogène avec des limites irrégulières. Une étude rétrospective récente a précisé quelques critères échographiques prédictifs de malignité tels que la présence de calcifications intra-lésionnelles et l'infiltration des tissus avoisinants. Par ailleurs, L'absence de vascularisation intra-tumorale, la forme ovoïde de la lésion avec une capsule épaisse ont une valeur prédictive négative, qui rendent la malignité peu probable [2, 4]. Les études ont montré que la sensibilité échographique, pour les carcinomes parathyroïdiens, varie de 50 à 90% [4]. La scintigraphie au Tc-99m-sestamibi est très utile pour la localisation de la tumeur, caractérise le tissu parathyroïdien anormal ou ectopique. Mais, elle ne fournit pas de renseignements quant à la nature bénigne ou maligne de la tumeur. Cependant, elle pourrait être utile aussi dans le diagnostic et la localisation des métastases ganglionnaires ou à distance des carcinomes parathyroïdiens [4]. Autres techniques d'imagerie peuvent être utiles, le couple TDM/IRM a une meilleure sensibilité par rapport à la scintigraphie au Tc-99m-sestamibi, mais une spécificité similaire [3]. Plusieurs constatations peropératoires ont été décrites, à travers la littérature, qui caractérisent le carcinome parathyroïdien. Ce dernier est souvent décrit comme une lésion lobulée, de consistance ferme à dure, entouré d'une capsule fibreuse blanc-grisâtre dense qui adhère fortement aux tissus adjacents et rend la tumeur difficile à disséquer des structures contigües ce qui fait du diagnostic de cancer très probable [13]. Chez notre patiente, c'était effectivement une formation lobulée, de consistance dure, infiltrant les tissus avoisinants. Comme plusieurs néoplasies endocriniennes, la distinction entre tumeur parathyroïdienne bénigne et maligne est difficile. En 1973, Shantz et Castleman, ont défini un ensemble de critères histologiques pour le diagnostic carcinome parathyroïdien sur la base d'une analyse de 70 patients atteints, à savoir, l'architecture trabéculaire avec des cloisons fibreuses denses responsables de l'aspect lobulaire, l'invasion capsulaire ou vasculaire, les atypies cytonucléaires et les mitoses [6, 14].

**La résection** Chirurgicale est le traitement de base du carcinome parathyroïdien [3-5]. Des métastases ganglionnaires cervicales sont retrouvées chez environ 25% des patients [5]. Le geste chirurgical doit inclure une exérèse de la tumeur parathyroïdienne, du lobe thyroïdien homolatéral, et un évidement des chaînes ganglionnaire prétrachéales et récurrentielles. L'évidement jugulocarotidien homolatéral est effectué si présence d'adénopathies manifestement métastatiques. La plupart des auteurs s'accordent qu'une résection incomplète est à haut risque de récidive et qu'une exérèse tumorale élargie est la meilleure option pour espérer la guérison [3, 4, 6]. Il est impératif de ne pas rompre la capsule parathyroïdienne lors de l'exérèse chirurgicale pour éviter l'ensemencement des cellules tumorales et la récidive locale. Le sacrifice du nerf laryngé inférieur (récurrent) n'est nécessaire que si ce dernier est macroscopiquement envahi [5, 9]. Le carcinome parathyroïdien n'est pas une tumeur radio-curable selon différentes publications. Par contre, l'irradiation du cou après chirurgie ou pour une récidive peut être utile pour un meilleur contrôle local [13, 15]. Wynne et ses collaborateurs [9], ont rapporté une guérison apparente (10 ans) d'un carcinome parathyroïdien localement invasif, qui a été obtenue après traitement par radiothérapie. En outre, six patients qui ont reçu une radiothérapie après résection chirurgicale incomplète ont été suivis pendant 12 et 156 mois, sans récidive locale. On déduit que la radiothérapie peut réduire le risque de récidive locale du carcinome parathyroïdien après chirurgie. Dans une étude récente, les auteurs ont recommandé une radiothérapie postopératoire de 40-50 Gy chez les patients à haut risque de récidive locale [16]. L'efficacité de la chimiothérapie dans le cancer de la parathyroïde n'a pas été démontrée dans la littérature [2, 6]. La morbidité et la mortalité lors du cancer parathyroïdien sont essentiellement liées à l'hypersécrétion de PTH et à l'hypercalcémie qui en résulte, plutôt qu'à l'extension tumorale elle-même. Ainsi, la prise en charge de l'hypercalcémie est impérative chez des patients ayant une tumeur locale non resecable ou des métastases généralisées [3]. Des diurétiques de l'anse induisant la calciurèse sont prescrits (furosémide) sous surveillance horaire de la diurèse et de la natriurèse. En cas d'insuffisance rénale oligoanurique, une épuration extrarénale est nécessaire. On dispose de plusieurs molécules dans le but est d'inhiber la résorption osseuse telles que les Bisphosphonates [2, 3].

### Le facteur pronostic le plus important dans le cancer parathyroïdien est l'exhaustivité de la résection tumorale

Les patients qui bénéficient d'une résection tumorale complète en monobloc, peuvent avoir des taux élevés de survie qui arrivent jusqu'à 90% à 5 ans et 67% à 10 ans. Les facteurs de mauvais pronostic sont la présence de métastases ganglionnaires au moment du diagnostic, de métastases à distance et les carcinomes type non-sécrétant [3]. Le risque de récidive locale et métastastique (pulmonaire, osseux, et hépatique) a été estimé entre 25 et 60% dans les 2 à 5 ans qui suivent la résection initiale [6, 16]. La plupart des récidives se manifestent dans les 3 premières années, mais des récidives au-delà de 20 ans ont été aussi rapportées d'où l'utilité d'une surveillance prolongée [6, 17]. Cette dernière comporte le dosage de la calcémie dont la surélévation témoigne d'une récidive ou d'une métastase, en plus de l'examen physique à la recherche d'une masse cervicale, d'une adénopathie ou d'une paralysie récurrentielle. Le traitement des récidives et des métastases est chirurgical lorsqu'il est possible et compatible avec l'état général du patient [6].

## Conclusion

Devant la présentation atypique d'une tumeur de la parathyroïde, le diagnostic de carcinome parathyroïdien est généralement établi sur la conjonction de signes cliniques, radiologiques, biologiques et histologiques. La gravité de cette pathologie est due à l'hypercalcémie sévère aggravant la mortalité et au risque de récidive et de métastases à distance justifiant la surveillance prolongée.

## Conflits d’intérêts

Les auteurs ne déclarent aucun conflit d'intérêts.
